# Revisiting the Role of the CXCL13/CXCR5-Associated Immune Axis in Melanoma: Potential Implications for Anti-PD-1-Related Biomarker Research

**DOI:** 10.3390/life13020553

**Published:** 2023-02-16

**Authors:** Magdalena Hoellwerth, Peter Koelblinger, Roland Lang, Andrea Harrer

**Affiliations:** 1 Department of Dermatology and Allergology, Paracelsus Medical University, 5020 Salzburg, Austria; 2 Department of Neurology, Christian Doppler University Hospital, Paracelsus Medical University and Center for Cognitive Neuroscience, 5020 Salzburg, Austria

**Keywords:** CXCL13, CXCR5, biomarker, melanoma, anti-PD-1 therapy, TLS

## Abstract

CXCL13 is a potent chemoattractant cytokine that promotes the migration of cells expressing its cognate receptor, CXCR5. Accordingly, T follicular helper cells and B cells migrate towards B cell follicles in lymph nodes, where the resulting spatial proximity promotes B cell/T cell interaction and antibody formation. Moreover, effector cells of the CXCL13/CXCR5-associated immune axis express PD-1, with corresponding circulating cells occurring in the blood. The formation of so-called ectopic or tertiary lymphoid structures, recently detected in different cancer types, represents an integral part of this axis, particularly in the context of its emerging role in anti-tumor defense. These aspects of the CXCL13/CXCR5-associated immune axis are highlighted in this review, which focuses on cutaneous malignant melanoma. Specifically, we elaborate on the role of this important immune axis as a possible ancillary target of immune checkpoint inhibition with anti-PD-1 antibodies in different therapeutic settings and as a potential source of predictive biomarkers regarding treatment efficacy.

## 1. Introduction

Melanoma is considered the most aggressive skin cancer due to its high propensity to develop metastases (https://seer.cancer.gov/statfacts/html/melan.html, accessed on 28 November 2022) [1]. Immunotherapy with checkpoint inhibitors has evolved into the preferred first-line therapy in the metastatic and adjuvant setting and is considered a milestone in cancer history [2].

Pembrolizumab and nivolumab are monoclonal antibodies acting as immune checkpoint blockers and have demonstrated clinical efficacy in the treatment of melanoma through inhibition of the programmed cell death protein one (PD-1) receptor [3,4,5]. Anti-PD-1 therapy is pivotal in the treatment of invasive melanoma; however, its clinical benefit is limited to a subset of patients and the extent of the clinical response strongly varies between patients [6,7]. While some patients show durable long-term treatment responses, others experience rapid tumor progression despite immunotherapy [7]. The strong individual differences accentuate the need for the development of biomarkers in this field, making it a hot topic in current research [1,8,9,10,11,12,13,14].

To achieve successful immunotherapy with immune checkpoint inhibitors in the adjuvant, neoadjuvant, and advanced settings in melanoma, an effective, adaptive, anti-tumor immune defense is a crucial prerequisite. According to the proposed primary mode of action, exhausted anti-tumor specific CD8+ T cells become reinvigorated through the blockage of PD-1, transforming them into functional defenses. However, accumulating evidence highlights the relevance of CD8+ T cell cooperation with B cells and CD4+ T cells and the formation of tertiary lymphoid structures (TLS) in nurturing adaptive immune cell interactions in inflamed and tumor tissue [15]. The CXCL13/CXCR5-axis plays a crucial role in orchestrating B and T cell interactions and has emerged as an important contributor to the formation of TLS [16,17].

Importantly, the key effector cells of the CXCL13/CXCR5-associated immune axis express PD-1, hence attracting increasing interest as secondary or ancillary targets of anti-PD-1 therapy in different cancer types. As circulating counterparts of these effector cells are detectable in blood, the effects of anti-PD-1 therapy on circulating immune cells are also worth exploring [18,19].

**Figure 1:**
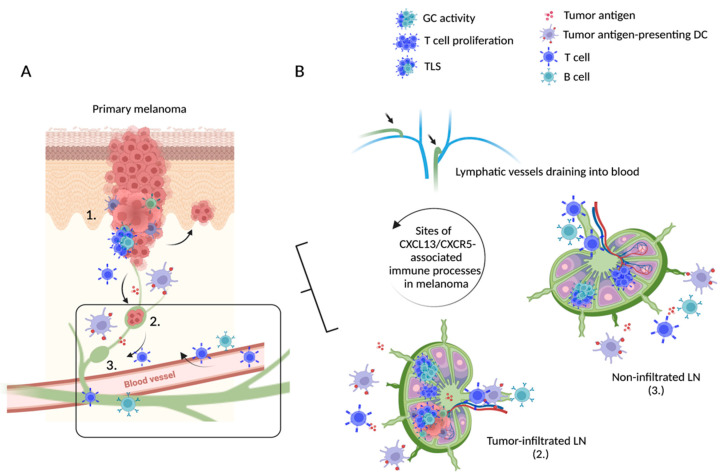
Overview of possible sites with CXCL13/CXCR5 activity in cutaneous melanoma. (**A**) At the site of invasive melanomas, tumor infiltration of immune cells may lead to the formation of more or less structured TLS (1). The subsequent tumor-specific immune response includes transfer and migration of tumor antigens, tumor-specific T cells, and tumor antigen-presenting DC to the associated draining lymph nodes. The extent of tumor infiltration of draining lymph nodes may vary (2, 3). Specifically primed anti-tumor immune cells migrate back to the primary tumor site via the efferent lymph and blood vessel. (**B**) Details regarding tumor-draining lymph nodes and circulating immune cells (black square): tumor antigens, tumor antigen-presenting DC, and anti-tumor effector T cells enter the tumor-infiltrated sentinel lymph node (2), triggering an anti-tumor GC response, associated T cell proliferation, and TLS formation within the affected LN. Tumor antigens, educated B and T cells, and tumor antigen-presenting DC travel via efferent lymphatic vessels to downstream LNs (3) where they further engage in mounting anti-tumor immune responses and education and proliferation of effector cells, which again circulate back to the site of the primary tumor. Abbreviations. TLS: tertiary lymphoid structures, GC: germinal center, DC: dendritic cells, LN: lymph node. We thank Tobias Moser for his support with the figure. Created with BioRender.com.

**Figure 2:**
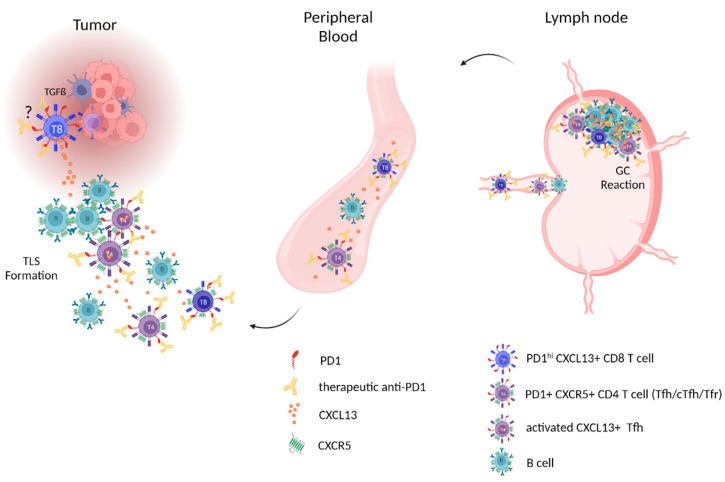
Model of PD-1-expressing CXCR5/CXCL13-associated immune effector cells in tumor tissue, peripheral blood, and lymph nodes representing possible targets of anti-PD-1 therapy effects. Left: question mark is indicating a TGF-ß-dominated immune suppressive tumor microenvironment as a possible trigger stimulating exhausted CD8+ T cells (with therapeutic anti-PD-1 on their surface) to produce CXCL13. The resulting CXCL13 gradient contributes to the recruitment of B cells, CXCR5+ PD-1+ CD8+, and CXCR5+ PD-1+ CD4+ T cells (with anti-PD-1 on their surface) from the blood (middle)—in particular Tfh cells, which beyond interacting with B cells also can contribute via CXCL13 production—and the formation of TLS. Right: tumor-specific GC response with anti-PD-1-bound Tfh and proliferating B cells in tumor-draining lymph nodes and the exit of educated B cells, and anti-PD-1-bound CXCR5+ PD-1+CD8 and CXCR5+PD-1+CD4 T cells via efferent lymphatic vessels into the blood. Abbreviations: PD-1: programmed cell death protein one; Tfh: T follicular helper cells, TLS: tertiary lymphoid structures. We thank Tobias Moser for his support with the figures. Created with BioRender.com.

This review summarizes our current understanding of the CXCR5/CXCL13 immune axis, its role in TLS formation within cancer tissue and the associated tumor microenvironment (TME), and its influence on anti-tumor immune defenses (summarized in Figure 1). Specifically, we propose that the CXCR5/CXCL13-axis may represent an additional target for anti-PD-1 therapy in cutaneous melanoma, that is relevant for treatment outcome as well as a potential source of predictive therapeutic biomarkers (summarized in Figure 2). 

### 1.1. The CXCL13/CXCR5-Axis and Secondary Lymphoid Organs—Focus on Lymph Nodes

CXCL13, a potent B cell chemoattractant, is expressed by stromal cells (predominantly by macrophages and follicular dendritic cells) and induces migration of B cells and particular T cell subsets towards B cell follicles within lymph nodes (LN) [20,21]. CXCR5 is the cognate receptor of CXCL13, expressed on B cells and follicular B cell helper CD4+ T cells (Tfh), which are professional B cell helper T cells [22,23,24]. Upon entry into LN, interaction with antigen-presenting dendritic cells can induce upregulation of CXCR5 on naïve T cells, thereby enabling their migration as pre-Tfh cells towards the B cell zone, where they further differentiate into Tfh cells [25]. When f is differentiated, Tfh express high levels of PD-1, Bcl6, and ICOS and produce cytokines such as IL-21, IL-4, and IL-10, which promote germinal center (GC) reactions [19,26]. Of note, PD-1hi-Tfh-cells themselves produce CXCL13 upon activation [27]. 

Spatial proximity within B cell follicles allows highly specific and productive interactions of B cells and Tfh cells, along with the formation of GCs, the main sites of B cell proliferation, differentiation, BCR affinity maturation, and antibody production, resulting in the generation of memory B cells, long-lived plasma cells, and high-affinity antigen-specific IgG antibodies [16,26]. Tfh cells have a fundamental role in the function and maintenance of the humoral immune system [16,21,28,29]. In addition to B cells and Tfh cells, additional CXCR5+-expressing lymphocytes exist, e.g., T follicular regulatory cells (T_FR_), natural killer T follicular helper (NK-Tfh) cells, follicular regulatory CD8+ T cells (CD8-Tfr), and follicular CD8+ T cells (Tf8). The presence of these cell types within B cell follicles and their contribution to the GC reaction is increasingly recognized [25]. Similar to conventional regulatory T cells, T_FR_ cells can regulate B cells and control germinal center reactions to prevent autoimmunity [30].

Higher levels of CXCL13 indicate an activated immune status and are observed in inflammation, vaccination responses, and autoimmune diseases [31]. With regard to cancer immunology, significantly higher CXCL13 mRNA expression was detected in draining compared to non-draining LN [15].

### 1.2. The CXCL13/CXCR5-Axis and Tertiary Lymphatic Structures/Ectopic Lymphoid Structures

At sites of enduring inflammation and persistent antigen exposure, such as chronically inflamed cancer tissue, infiltrating immune cells can form TLS, structured aggregates that resemble LN. TLS can form in any tissue affected by prolonged inflammation and can resolve temporarily upon successful antigen elimination [17,32]. TLS occur at different stages of maturation, depending on their arrangement and structural compartmentalization of participating immune cells. While immature TLS are poorly structured cell clusters containing loose aggregates of B cells and T cells, mature TLS are characterized by a LN-like architecture with segregated B cell follicles and T cell zones [17,33]. They can even feature high endothelial venules (HEV), highly specialized blood vessels for lymphocyte entry from blood into lymphoid tissue [34]. Although TLS share structural similarities, such as separated B and T cell areas and the particular plump endothelium, an essential difference is the sheathing, as TLS lack a capsule [17,33,35].

The initial trigger leading to the development of TLS remains unclear. However, different cytokines and chemokines are involved, with CXCL13 and CXCR5 proposed to be essential [36,37,38,39]. In the organization of TLS within tumors, different contributors are involved ranging from Tfh cells, B cells, to Tf8 cells—cells that lure other immune cells to the site of aggression [25]. Peripheral CXCR5-negative Tfh-like cells and Tfh that have migrated into the tumor are potent CXCL13 producers, attracting more B cells and CXCR5+ T cells to the TME [40]. Thus, CXCL13-producing cells play an essential role in the organization and genesis of TLS [31,41], generating durable memory cells [27,42]. Moreover, exhausted CD8+ T cells are capable of producing CXCL13, which in turn attracts Tfh cells and is involved in the formation of TLS [15].

As in secondary lymphoid organs, GC reactions also take place in mature TLS, with clonal expansions of B cells and humoral antibody-producing zones. However, antibody responses have only been described in mature TLS [17,32]. The generated humoral and cell-mediated responses also play important roles in the anti-tumor response and are considered part of the tumor defense arsenals [43].

### 1.3. The CXCL13/CXCR5-Axis in Peripheral Blood

Circulating blood CD4+CXCR5+ cells have been identified as the peripheral form of Tfh, here termed cTfh. CXCR5 is considered a reliable marker to detect Tfh in the periphery, as it was found to be the most stable among Tfh cell markers [44]. cTfh have memory-like properties and are largely found in a resting state, circulating in the periphery in order to patrol for antigens [45]. 

cTfh can be further divided into three subgroups namely T_FH1_, T_FH2_, and T_FH17_. T_FH1_ is recognizable as CXCR3+CCR6-, T_FH2_ as CXCR3-CCR6-, and T_FH17_ as CXCR3-CCR6+, respectively. Both T_FH2_ and T_FH17_ are considered potential helpers of naïve B cells and trigger antibody production. T_FH1_ are not capable of triggering a response in naïve B cells. However, activated T_FH1_, which are ICOS+PD-12+CCR7lo, participate in recall immune response and the conversion of memory B cells into plasma B cells [19,23,46]. 

In infection and vaccination studies, increased blood CXCL13 levels have been found and correlated with levels of antibody production and numbers of activated cTfh. This led to the idea that CXCL13 may act as an indicator of B cell-related immune activity. In particular, it has been proposed as a soluble blood biomarker for GC activity [27]. 

In cancer immunology, the search for reliable peripheral biomarkers is also intense, given the ease of sample collection and the ability to monitor treatment pharmacodynamic immune activity over an extended period of time. In contrast to tumor tissue, where surgical collection is usually restricted to the time points of initial tumor diagnosis and progressive disease, blood is easily accessible and can be obtained at any time. The CXCL13/CXCR5-axis, with its prominent role in launching adaptive immune responses and its circulating soluble and cellular components, could be an additional promising resource for anti-cancer biomarker research in immunogenic tumors, such as melanoma.

## 2. CXCL13/CXCR5-Associated Immune Activity and Anti-Tumor Immunology in Melanoma

Due to its high mutational burden [47], melanoma is considered an immunogenic tumor and consequently represents one of the cancer entities most sensitive and responsive to immunotherapy [48,49]. Within the tumor-specific immune response, malignant cells are supposed to be recognized and destroyed by CD8+ cytotoxic T cells. Consequently, in cutaneous melanoma, the presence and quantity of infiltrating CD8+ lymphocytes represent favorable prognostic factors [50,51]. 

In addition, there is a correlation between mutational burden and neoantigen load. Hence, in metastatic melanoma a high neoantigen count correlates with improved clinical outcome among immunotherapy patients, whereas a low immune infiltration is considered a poor prognostic factor [49,52].

However, at the same time, constant interaction with the TME through intra- and extracellular signaling enables melanoma to undergo immune editing in order to evade recognition and elimination by the immune system [53,54]. In addition, persistent exposure to tumor antigens causes CD8+T cells to enter a state of exhaustion, which is marked by upregulation of inhibitory checkpoint molecules (such as PD-1 or LAG-3). The exhaustion process in CD8+ T cells happens gradually, with reduced IL-2 signaling and TNF-α production, accompanied by a shutdown of cytotoxic effects [13,53,55]. Importantly, exhaustion is a not a binary state but involves many transitional functional states [56]. According to our current understanding, so-called progenitor exhausted CD8+ T cells are most responsive to anti-PD-1-mediated reinvigoration. Thus, the presence of these progenitor cells is considered a prerequisite for successful immune checkpoint inhibition therapy [15]. T cell exhaustion represents a highly complex process and is described in detail by van der Leun et al. [57].

For the above reasons, most scientific efforts with regard to cellular tumor control have primarily focused on therapeutic responses regarding cytotoxic T cell activity [3]. However, the extent to which CD4+ T cells and B cells are involved in anti-tumor immune defense is increasingly gaining attention. Given the central role of PD-1 inhibition in cancer immunotherapy, this also pertains to PD-1-expressing components of the CXCL13/CXCR5-axis [53].

### 2.1. Current Evidence of CXCL13/CXCR5-Related Immune Activity in Early and Advanced Melanoma

In melanoma, high chemokine scores in analyzed gene databases have been linked to histopathological detection of increased peritumoral lymphocytic infiltration, considered as TLS [58]. Metastatic melanoma patients with a high CXCL13 expression in tumor tissue showed a better overall survival [59]. In a systematic review of 69 studies including 15 cancer entities, Wouters et al. were able to reveal a predominantly positive correlation between the occurrence of B cells in cancer and patient survival. In only 9.3% of the analyzed studies was the presence of CD20+ TIL considered a negative prognostic predictor. In malignant melanoma explicitly, the presence of B cell signatures correlated with increased overall survival and better outcome in mRNA sequencing analyses (HR 0.86) [60]. Cipponi et al. described TLS in metastatic melanoma tissue, but were unable to detect mature TLS in primary melanoma samples [39]. In contrast, Wagner et al. analyzed the immunohistochemical staining of 103 melanoma tissues and reported the presence of TLS in primary melanoma, although a large fraction were at an immature stage [61]. TLS were detected in the vast majority of metastatic tissue sections (81.2%), and it is particularly interesting that all lymph node metastases contained secondary follicular TLS. TLS were associated with a high infiltrate of dendritic cells, B cell aggregates, and HEV, and their presence correlated with the status of disease, with very low TLS density observed in primary lesions, and increasing density with advancing disease. The presence of B cells was regarded as an indicator of a good therapeutic response, highlighting the necessity and importance of research focusing on the CXCL13/CXCR5-associated immune axis in primary and metastatic melanoma [61].

With regard to the development of TLS, a hypothesis from an animal model was adapted. It addressed the fact that in the context of immunoediting and immunoescape, tumors generate a TGF-ß-dominated milieu, which triggers the production of CXCL13 in exhausted CD8+ T cells [15].

High CXCL13 levels, in turn, lead to the recruitment of CXCR5-positive Tfh and B cells to, and their aggregation in, the tumor site. Once there, Tfh cells contribute to rescuing CD8+T cells via IL-21, a cytokine responsible for the maintenance of CD8+T cell function, including their cytotoxic effects on the immune system and preventing them from final exhaustion [15]. 

The anti-cancer immune effects of Tfh cells are manifold, as they proposedly promote the development of TLS, consisting of CD8+ T cells, macrophages, and natural killer cells. They can also engage with B cells, promoting their local activation and tumor-specific antibody synthesis. In this way, an anti-tumor defense is generated in the immediate local vicinity of the tumor instead of further away in the LN [15]. Accordingly, higher levels of Tfh cells, along with infiltration of Th1 cells in solid tumors, are associated with improved patient survival in various cancers [62]. 

In this context, Hoch et al. investigated the chemokine milieu in metastatic melanoma by multiplex mass cytometry analyses, providing detailed spatial information regarding the interaction of tumor and immune cells down to the level of cyto- and chemokines. They found that tumors with a dense immune cell infiltrate revealed a higher expression of multiple chemokines and demonstrated a better responsiveness to immunotherapy. Above all, CD8+ T cells were present in melanomas with a high mutation load, and they did not only express CXCL13 but also exhaustion markers (e.g., PD-1). This observation again reinforces the importance of these cells and could indicate that they still retain their function [63].

### 2.2. Anti-PD-1 Therapy and the CXCL13/CXCR5-Axis

Anti-PD-1 antibody-based immunotherapy was first approved in 2015 for the treatment of metastatic melanoma. The predominant mode of action of the PD-1 blockade was considered to be reinvigoration of tumor-specific CD8+ cells in the tumor tissue [64,65]. In 2018, PD-1 antibodies were also approved for the adjuvant treatment setting, in which immunotherapy is initiated after complete surgical resection of the primary melanoma and associated LN or distant metastases. The rationale for adjuvant therapy is to prevent melanoma recurrence by treating clinically non-detectable metastases, i.e., circulating tumor cells, that still may be present [4]. Significantly improved recurrence-free survival with adjuvant immunotherapy compared with the placebo has demonstrated that efficacy of anti-PD-1 therapy does not necessarily require the presence of macroscopic tumor tissue, but also depends on mechanisms such as immune surveillance and the contribution of immune cells circulating in blood or lymph vessels. 

With increasing awareness of anti-PD-1 treatment effects beyond the site of the primary tumor or distant metastases, the importance of tumor-draining LN was recognized. Here, immune cells presenting tumor-derived antigens encounter naïve T and B cells, which upon activation proliferate and differentiate into tumor-specific effector cells that enter the TME via the bloodstream to replenish/refurbish the anti-tumor immune response. Within tumor-draining LNs, immune cells (apart from CD8+ T cells) are substantially involved in the process of immune activation and may represent side targets of anti-PD-1 treatment. For example, Tfh cells in B cell follicles frequently show surface expression of PD-1; hence, they are interacting with therapeutic antibodies. Moreover, the recently detected progenitor exhausted CD8+ T cells were described to express CXCR5, indicating another association with the CXCL13/CXCR5-axis [66]. This specific CD8+ T cell subset was found to be tumor antigen specific [67] and to proliferate in response to the PD-1 blockade, hence representing a proposed main target of anti-PD-1 therapy and an indicator of increased therapeutic responsiveness [56,68].

Despite the different off-site effects of anti-PD-1 treatment outlined above, recent neoadjuvant therapeutic approaches—in which anti-PD-1 antibodies are administered for a limited period of time prior to surgical removal of macroscopic (LN) metastases—have shown that, compared to the adjuvant treatment scenario, efficacy is superior when an intact TME is present at the time of treatment initiation. This superiority was most clearly demonstrated in the SWOG S1801 trial comparing standard-of-care adjuvant pembrolizumab in stage III melanoma with three cycles of neoadjuvant pembrolizumab treatment followed by surgery and adjuvant treatment. Despite a relatively low pathological complete response rate of 21%, event-free survival in stage IIB-IV melanoma patients was significantly improved in patients receiving neoadjuvant treatment at the two-year time point (72 vs. 49%, *p* = 0.004). Combined immunotherapeutic approaches with nivolumab plus ipilimumab or relatlimab have demonstrated markedly higher complete pathological response rates of approximately 60% in the neoadjuvant setting, suggesting superior efficacy [69,70]. In general, pathological response after neoadjuvant immunotherapy has been identified as a useful biomarker predictive of recurrence-free survival. Pathologic responses have been associated with the accumulation of exhausted CD8+ T cells in the tumor three weeks after first anti-PD-1 administration, with reinvigoration of T cells in the blood observed as early as one week post treatment [14]. In the near future, neoadjuvant treatment enabling paired investigation of tissue and blood samples will allow for intensive research regarding potential predictive biomarkers beyond the pathologic response rate; among others, components of the CXCL13/CXCR5-associated immune axis will be of interest.

### 2.3. Implications for Biomarker Research: Tissue and Peripheral Blood

In melanoma, a variety of different immune phenotypes have been identified, potentially contributing to the heterogeneous response observed with the immune checkpoint blockade [19]. It is therefore necessary to focus on immune cell populations beyond CD8+ T cells, particularly on CD4+ T-helper cells and B cell subsets.

In addition, the presence of helper CD4+ T cells, Tfh, and B cells is positively associated with patient survival [51], and it must be emphasized that certain CXCR5+ Tfh cells are recognized as promoters of CXCL13/CXCR5-associated immune activity, with subsets of cells proposed to be responsive to anti-PD-1 therapy [71]. With the knowledge of the many off-targets of anti-PD-1-therapy, such as CXCL13/CXCR5-associated components, it will be essential to incorporate them into a holistic biomarker research approach for predicting treatment response.

It is also recognized that a high neoantigen burden with a dense tumor microenvironment and upregulation of IFN-γ-related genes are positive predictors of a good response to immunotherapy [11]. 

An inflammatory subtype marked by a high Th17 and Th1 gene signature is seen as a prerequisite for good balance with improved progression-free and overall survival. Across all identified immune types, a high lymphocyte signature and increased Th17 cells were associated with improved overall survival, whereas Th1 cells and Th2 cells were associated with worse and mixed effects regarding overall survival, respectively [71]. These findings should now be further investigated in the CXCR5 CD4+ subtypes, which can also be subcategorized into Th17, Th1, and Th2, including clonality and melanoma-specificity in the TME and periphery, since these subpopulations are often affected as side targets [19].

Monitoring the blood enables an examination of these cells in detail. Moreover, emerging neoadjuvant therapy regimens create new opportunities for tissue-based predictive biomarker research. This will allow important insights into anti-PD-1 treatment-related effects on the anti-tumor defense, functional regulation, and networks, such as the interplay of adaptive and innate CXCL13/CXCR5-associated components—from CXCL13-producing and attracting cells to various effectors that express a wide array of functions, ranging from cytotoxic and helper functions to humoral responses.

Moreover, the knowledge of the composition of TIL and circulating cells, with regard to the propensity for reinvigoration via CXCR5+ progenitor exhausted CD8+ T cells, may allow more accurate estimates of the outcome. 

The high potential and feasibility of monitoring circulating CXCL13/CXCR5-associated effectors has already been illustrated by Herati et al., who demonstrated an increase in circulating Tfh and B cells and CXCL13 serum levels in response to influenza vaccination in a subset of anti-PD-1 treated patients [72].

Similarly, in mature TLS, the presentation of tumor-derived antigens by B cells leads to enhanced T cell activity, indicating a more efficient response to immunotherapy [32]. Melanoma patients responding to PD-1-immunotherapy showed a marked difference in the gene expression of B cell markers compared to non-responders [73]. Werner et al. created a reproducible nomenclature to accurately measure TLS in melanoma via immunohistochemistry [61]. Thus, a tool has been established to further determine complex relationships between cell populations and their impact on disease activity, particularly in the context of response or failure of immunotherapy, which may serve as basis for further studies.

## 3. Conclusions

CXCL13/CXCR5-associated immune processes are fundamentally involved in anti-tumor defense, and the effector cells of the axis are all but irrelevant side-targets of anti-PD-1 therapy. In the tumor environment, TGF-ß acts as the major immunosuppressive cytokine and signals tumor progression, at the same time initiating CXCL13 expression in exhausted CD8+ T cells, synonymous for an escalation of the anti-tumor immune response [15]. The recruited Tfh and B cells, in turn, help to reinforce anti-tumor immunity by promoting inflammation, recruiting further immune cells, and by locally establishing more or less structured but functional lymphoid tissue. CXCR5 expression, reported on progenitor exhausted CD8+ T cells, links this potential primary target of anti-PD-1 therapy to the CXC13/CXCR5-axis. Furthermore, the PD-1 expression on Tfh and T_FR_ cells and their propensity to expand in response to anti-PD-1 therapy must be addressed. 

For melanoma, an immunogenic tumor with limited responsiveness to anti-PD-1 therapy, the necessity to further dissect the relevance and contribution of this immune axis to anti-tumor defense is obvious. Beyond identifying prognostic and predictive biomarkers regarding treatment response and survival, increasing insight may further support emerging treatment concepts, such as neo-adjuvant therapy. Strengthening the anti-tumor defense in both the tumor per se and within the tumor-draining LN as a core site for replenishing TIL [67] appears to be a concept worth pursuing. Additional independent defense mechanisms in the form of structured TLS may become increasingly important.

It can be concluded that the CXCL13/CXCR5-chemokine axis functionally connects B and T cell processes, underlined by the detection of TLS in tumor tissue. Both cellular and humoral components of the CXCL13/CXCR5-associated immune axis can be detected/assessed in blood, representing a source for potential biomarker candidates for monitoring treatment response to the anti-PD-1 checkpoint blockade. 

## Data Availability

Not applicable.
